# Estimation of Quasi-Stiffness of the Human Knee in the Stance Phase of Walking

**DOI:** 10.1371/journal.pone.0059993

**Published:** 2013-03-22

**Authors:** Kamran Shamaei, Gregory S. Sawicki, Aaron M. Dollar

**Affiliations:** 1 Department of Mechanical Engineering and Materials Science, School of Engineering and Applied Science, Yale University, New Haven, Connecticut, United States of America; 2 Joint Department of Biomedical Engineering, North Carolina State University and University of North Carolina at Chapel Hill, Raleigh, North Carolina, United States of America; University of Rochester, United States of America

## Abstract

Biomechanical data characterizing the quasi-stiffness of lower-limb joints during human locomotion is limited. Understanding joint stiffness is critical for evaluating gait function and designing devices such as prostheses and orthoses intended to emulate biological properties of human legs. The knee joint moment-angle relationship is approximately linear in the flexion and extension stages of stance, exhibiting nearly constant stiffnesses, known as the quasi-stiffnesses of each stage. Using a generalized inverse dynamics analysis approach, we identify the key independent variables needed to predict knee quasi-stiffness during walking, including gait speed, knee excursion, and subject height and weight. Then, based on the identified key variables, we used experimental walking data for 136 conditions (speeds of *0.75–2.63 m/s*) across 14 subjects to obtain best fit linear regressions for a set of general models, which were further simplified for the optimal gait speed. We found R^2^ > 86% for the most general models of knee quasi-stiffnesses for the flexion and extension stages of stance. With only subject height and weight, we could predict knee quasi-stiffness for preferred walking speed with average error of *9%* with only one outlier. These results provide a useful framework and foundation for selecting subject-specific stiffness for prosthetic and exoskeletal devices designed to emulate biological knee function during walking.

## Introduction

Mechanisms that can emulate human-like biomechanics are essential for robust performance of a number of engineered locomotion systems including anthropomorphic bipedal robots [1], [2], lower-limb wearable exoskeletons [*3*]–[*6*], and biologically-inspired prosthetic limbs [*7*]–[*10*]. Ideally, successful emulation of human locomotion in artificial systems is built upon a foundation of simple models (theoretical or empirical) that can accurately characterize the normal mechanical behavior of the human limb during gait [*11*]–[*13*].

Researchers have proposed theoretical models of varying complexity for the whole limb that can generate human locomotion [1], [*13*]–[*21*]. From the experimental side, it is possible to characterize the kinetic and kinematic behavior of the joints using data captured from humans in a gait laboratory [*22*]–[*24*]. It is also possible to study the force generating capabilities [25] as well as the passive and active stiffness of the joints using system identification techniques that employ statistical analyses and experimental data [*26*]–[*28*]. A common finding from all of these approaches is that compliance (i.e. springy limb behavior) plays a central role in shaping human motion. Design of assistive devices intended to mimic human behavior requires knowledge of how individual joints behave during locomotion tasks. Most reports of knee stiffness in the literature are for experiments performed under highly controlled laboratory conditions [*25*]–[*27*], making them difficult to extend to describe the knee behavior during locomotion in more general terms.

Recently, the concept of quasi-stiffness or “dynamic stiffness” [*30*]–[*38*] has been explored to characterize the spring-like behavior of lower-limb joints. The quasi-stiffness is defined as the stiffness of a spring that best mimics the overall behavior of a joint during a locomotion task. It can be estimated using the slope of the best linear fit on the moment-angle graph of the joint [*30*]–[*38*]. One should note that the quasi-stiffness of a joint explains how a joint functions during a locomotion task or phase, distinguishing it from the passive and active stiffness of a joint defined as a specific function of angle and time [26], [27]. The concept of quasi-stiffness applies particularly well to the knee joint during stance phase of walking, where a substantial moment is applied to compliantly support the body weight. This compliance was originally considered a determinant factor in reducing the vertical travel of center of gravity of the body [39], and later shown to play a major role in shock absorption [*40*]–[*42*]. Applying a preliminary quasi-stiffness analysis revealed a nearly linear spring-like behavior that changes with both gait speed and load carriage [34]. Indeed, a simple spring-like approximation of knee performance leads to much simpler mechanical designs of assistive devices, leading to greater robustness, lower cost, lighter weight, and higher shock tolerance.

The overall goal of this study was to establish a series of statistical models based on theoretical analysis and experimental data to characterize spring-type behavior of the knee in stance for adult humans spanning body size (height and weight) across a range of walking speeds. A well-developed general model of knee joint quasi-stiffness during walking promises to aid in diagnosis of musculoskeletal dysfunction and the development of biologically-inspired assistive devices (orthoses and prostheses) to improve mobility. For the latter applications, the stiffness of the knee joints will often need to be chosen in advance for specific cases or in a real-time form for employing a more complex active impedance control (e.g. [43]), such as recent efforts towards compliant stance-control knee orthoses currently being pursued by the authors [34] or compliant knee prostheses [43]. For these applications, generalized biomechanical models that can explain subject-specific variability of the behavior of lower extremity joints will be critical for sizing devices (e.g. choosing spring stiffness) to individual users.

We begin this paper with a description of the modeling and data collection methods used in the study, including an inverse dynamics analysis to obtain a generic expression for the knee moment from which we identify a subset of independent factors that can describe the quasi-stiffnesses of the knee during stance phase. We use an experimental data set (136 conditions across 14 subjects) spanning a substantial range of body size and gait speed of human adults to fit coefficients to these factors and present a series of general-form statistical models for quasi-stiffnesses that can account for the variability of the behavior of the knee among subjects based on body and gait parameters.

There are occasions where more simplified models that are primarily based on the body parameters might be favorable. This includes models for the design of compliant prostheses and orthoses, and exoskeletons for the knee that are versatile enough to perform well over a range of speeds around the energetically optimal gait speed. In order to apply the general-form models (described above), the magnitude of excursion for the knee and the speed as the gait parameters must be known. However, there are occasions where knee kinematics cannot be easily and repeatedly characterized (e.g. spinal cord injury patients), or where it would be undesirably time-consuming or expensive (such as in a prosthetist choosing a prosthesis stiffness for a specific patient). Accordingly, we also develop stature-based models that predict the knee quasi-stiffnesses at the optimal gait speed and for mean values of the knee excursion across the data.

## Methods

### Knee Phases of Motion in a Gait Cycle

**Figure 1:**
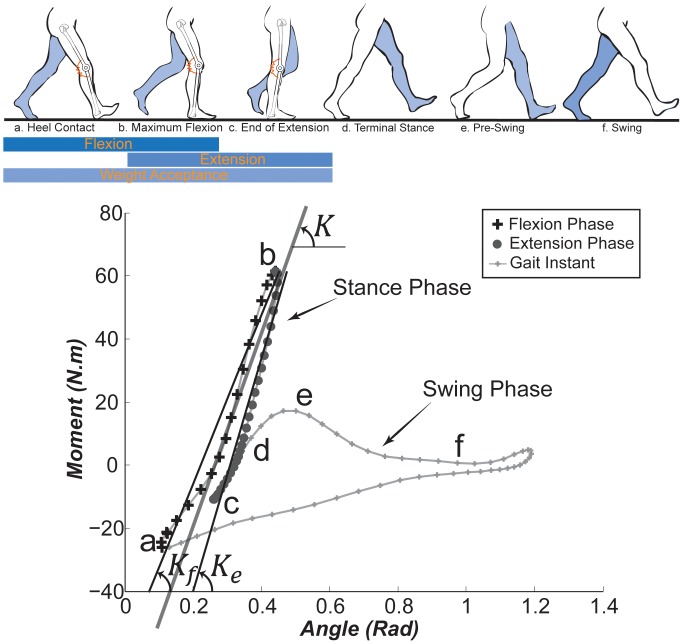
Knee moment vs. angle curve for a representative subject walking at 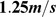. Letters a-f on the graph correspond to the poses shown during a typical walking cycle (top, schematic timing is adapted from [60]). Quasi-stiffness is calculated based on the slope of the best line fit to the moment-angle curve of a*–*b for the flexion stage (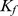), and b*–*d for the extension stage (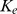) of the weight acceptance phase (a*–*d). The average of these two quasi-stiffness values is defined as the quasi-stiffness of the weight acceptance phase (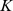).

To evaluate knee joint quasi-stiffness, we first divide the gait cycle into stance and swing phases (schematically shown in Fig. 1, top). The stance phase can be further divided into two sub-phases including a weight acceptance phase (consisting of the initial contact, loading response, and mid-stance phases) and a stance termination phase (consisting of the terminal stance and pre-swing phases) [44]. This study centers on the weight acceptance sub-phase (Fig. 1, top a*–*c). In this phase, the knee undergoes a flexion stage (a*–*b) and an extension stage (b*–*c) while supporting body weight. Exhibiting a shock damping mechanism [40], [42], the knee applies a large moment in the weight acceptance phase [45]. Accordingly, the knee is highly prone to collapse at this stage without proper action of the musculoskeletal system or external assistance (a problem that exists in patients with musculoskeletal disorders such as spinal cord injury and stroke). Contrary to the stance leg, the swing leg approximately undergoes a ballistic movement [21] that does not demand considerable muscular effort.

#### Terminology: Quasi-Stiffness and Angular Excursion of the Knee

We define the quasi-stiffness of the flexion stage (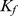) and extension stage (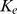) as the slopes of the lines fit on the moment-angle graph of the knee in the corresponding stage (see Fig. 1, bottom). We also introduce the quasi-stiffness of the entire weight acceptance phase (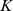) as the average of 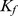 and 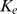. Alternatively, 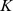 can be introduced as the slope of a line fit on the moment-angle graph of the weight acceptance phase. However, since the extension stage is more prolonged in time, the slope of the fit is highly affected by the behavior of the knee in that stage. We obtain the magnitude of excursion of the knee in the flexion stage (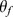) and extension stage (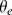) by subtracting the initial angle from the final angle in that particular stage. Using an averaging similar to the definition of 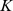, we define the knee excursion in the weight acceptance phase (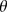) as the mean value of 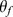 and 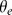.

### Identifying the Model Parameters and Form of Fits

We used a generalized, analytical inverse dynamics approach to derive an equation for the knee joint moment during human gait. The detailed analysis is documented in Appendix S1, Fig. S1 and Table S1. Briefly, we considered subject body weight (*W*) and height (*H*) as the body parameters, and walking speed (*V*), and magnitude of knee flexion (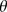) as the gait parameters. The approach is summarized as follows: 1) Simplify the general equation of the knee moment for the instant of maximum flexion in the weight acceptance phase of the gait (Fig. 1, point b) and extract the knee moment in the sagittal plane (*X-Y*), and 2) Extract theoretical model-forms by investigating the terms of the equation for the knee moment on the sagittal plane and correlate them with body and gait parameters.

The inverse dynamics analysis outlined in Appendix S1 suggests the following equation for the knee moment:



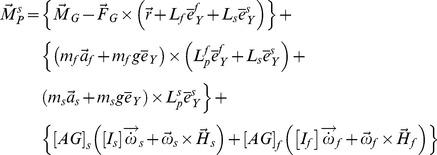
(1)


Parameter definitions for equation (1) and the equations that follow are listed in Table S1.

First, we simplify this equation for the instant of maximum flexion in the weight acceptance phase of the gait (Fig. 1, point *b*). At this instant, the ground reaction force (i.e. the force applied on the foot from the ground, GRF) shows a maximum magnitude for normal walking on a level ground. Moreover, since the ground reaction moment (i.e. the moment applied on the foot from the ground) is substantially smaller than the knee moment, we neglect it (i.e. 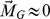). When the knee is maximally flexed in the stance phase, the support foot and shank segments are instantaneously nearly stationary (i.e. 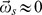 and 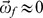). At this instant, the support limbs are dramatically loaded to propel the rest of the body. Thus, we assume that the effect of linear and angular acceleration of the support foot and shank is negligible compared to that of the rest of the body (i.e. 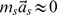 and 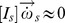, and 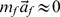 and 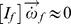). We further neglect the effect of the weight of the support limbs (i.e. 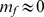 and 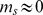). Applying these approximations in equation (1) results in the following expression for the knee moment:



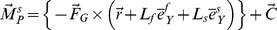
(2)where, 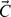 reflects the effect of the neglected terms. After our assumptions are applied, the analysis resides in a pseudo-static state which is valid for the instant of maximum moment in stance. We obtain the sagittal-plane component of the knee moment at the instant of maximum moment (point *b* in Fig. 1) from equation (2) as:
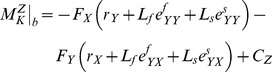
(3)where, 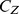 is the 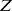-component of 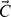 and 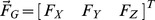. One should notice that 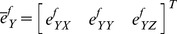, 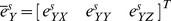, and 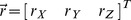. 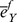 is assumed to be constant because the foot is instantaneously stationary when the knee is maximally flexed during the weight acceptance phase. We assume 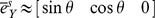, provided the leg moves only on the sagittal plane with the knee slightly flexed. Considering the small amount of flexion in normal walking we assume 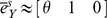. Anthropometric relationships imply that 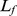 and 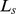 are proportions of 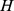 [46]. Also, it has been shown that center of pressure (COP) tends to lay underneath the ankle at the instant of maximum flexion in stance [47]. Therefore, 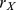 and 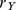 would be correlated with 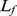, and hence with 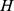. Therefore:



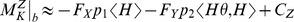
(4)where, in its general case, 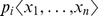 denotes an arbitrary first-order polynomial of 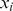
’s. Previous research has shown that the peaks of the normalized GRF (especially the peaks of vertical and anterior-posterior components in the stance phase) are correlated with the gait speed for normal walking on level ground [48]. In other words, at the instant of maximum moment in the weight acceptance phase we have:



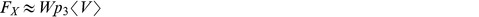
(5-a)




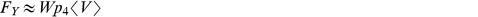
(5-b)


Applying equations (5-a) and (5-b) in equation (4) results in:



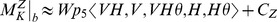
(6)


Assuming the knee behaves nearly linearly in the weight acceptance phase of the gait [34]:



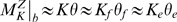
(7)


Combining (6) and (7) constitutes the following analytical forms for the quasi-stiffness of the knee in the weight acceptance phase, and its flexion and extension stages:



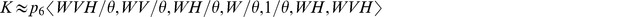
(8-a)




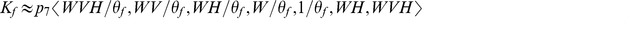
(8-b)




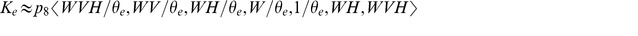
(8-c)


These equations suggest that, in its most general form, 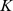 could be modeled by a first order polynomial of 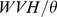, 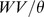, 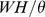, 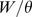, 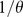, 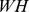, and 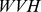 (and a function of only *V*, 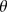, *H*, and *W*); and similarly for 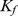 and 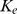.

### Experimental Protocol, Data Extraction and Statistical Analysis

We extracted knee joint angle and moment data from 14 subjects including unimpaired male and female adults spanning a reasonably wide range of weight (*67.7–94.0 kg*) and height (*1.43–1.86 m*). These data were provided to us by other researchers from previous studies from two labs:

1. Nine subjects (subjects 1 to 9 in Table 1) each walking at four different speeds on a treadmill at Human PoWeR Lab, NC State University. Data were taken under the IRB approval of University of North Carolina at Chapel Hill as detailed in [24]. The original paper reports data for ten subjects. However, we excluded the data from one subject who had an incomplete data record. Details on subject consents, collection protocols and data analysis for this subject group can be found in [24].
2. Five subjects (subjects 10 to 14 in Table 1) each walking at twenty different speeds on level ground at Biomechanics Lab, East Carolina University. The general procedures used to obtain the ground reaction force, sagittal plane knee joint angular position and torque are described in detail elsewhere [29]. We detail here the specific procedures relevant to the purpose of this study. All participants read and signed an informed consent form approved by the University Institutional Review Board at East Carolina University. Using a 15 m walkway, force platform (AMTI, Watertown, Ma) and eight camera motion capture system (Qualisys, Gothenberg, Sweden), three dimensional ground reaction force and linear position data describing the right lower extremity and pelvis were obtained from each participant during 20 walking trials of different velocities ranging from 1.01 to 2.63 ms^-1^. Each participant was initially tested at a self-selected, moderate walking speed the mean of which was 1.63 ± 0.03 ms^-1^. Subsequently, the 19 remaining trials per participant were collected in an approximately random order of walking velocities. Participants were instructed to walk at various speeds with instructions such as, “walk at a moderately fast pace,” “walk at a very slow pace,” and “walk at your fastest pace.” The mean walking velocity for all trials was *1.77 ± 0.36 ms^−1^*. All participants had similar minimum and maximum walking velocities and therefore similar ranges of walking velocities. Additionally, the 20 walking velocities for each participant, were moderately evenly distributed through the range of velocities from slowest to fastest velocities. Qualisys Track Manager and Visual 3D software (C-Motion, Gaithersburg, Md) were used to calculate the knee joint angular position and torque through the stance phase of walking in each trial from the linear position and ground reaction force data.

**Table 1:** Details on Subjects and Experimental Trials used for Regression Fits.

Subject	Gender	#Trial	*W*	*H*	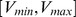	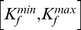	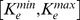	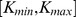	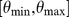	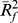	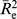	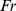 *when* 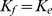
1‡	M	4	92.3	1.86	[0.75,2.00]	[284,376]	[283,297]	[330,390]	[6], [22]	90	96	0.174
2‡	M	4	68.4	1.70	[0.75,2.00]	[141,225]	[223,255]	[186,233]	[12], [25]	89	95	0.191
3‡	M	4	65.6	1.65	[0.75,2.00]	[155,266]	[221,261]	[198,244]	[11], [28]	90	94	0.197
4‡	M	4	94.0	1.86	[0.75,2.00]	[326,478]	[361,556]	[344,517]	[6], [17]	92	95	0.174
5‡	M	4	68.1	1.72	[0.75,2.00]	[182,255]	[291,582]	[273,382]	[7], [22]	91	84	0.189
6‡	F	4	57.7	1.43	[0.75,2.00]	[145,255]	[197,291]	[187,255]	[11], [19]	91	97	0.227
7‡	F	4	63.1	1.45	[0.75,2.00]	[114,185]	[81,308]	[98,231]	[9], [24]	87	95	0.224
8‡	F	4	65.7	1.75	[0.75,2.00]	[161,456]	[237,739]	[278,450]	[6], [17]	93	94	0.185
9‡	F	4	75.9	1.80	[0.75,2.00]	[237,393]	[292,378]	[291,343]	[10], [22]	93	94	0.180
10†	M	20	85.7	1.74	[1.26,2.43]	[236,569]	[244,342]	[279,422]	[13], [20]	99	96	0.254
11†	M	20	79.2	1.82	[1.38,2.25]	[227,414]	[258,331]	[256,343]	[16], [23]	98	98	0.246
12†	M	20	62.1	1.64	[1.04,2.29]	[119,379]	[144,278]	[155,269]	[6], [17]	98	96	0.234
13†	M	20	62.0	1.62	[1.01,2.44]	[163,351]	[143,188]	[158,263]	[11], [24]	99	95	0.262
14†	M	20	75.1	1.77	[1.30,2.63]	[248,745]	[210,384]	[260,565]	[12], [23]	99	96	0.247
		**Mean**	**72.5**	**1.70**	**1.68**	**304**	**263**	**284**	**16.5**	**93**	**94**	**0.213**
		**SD**	**11.5**	**0.04**	**0.42**	**114**	**91**	**78**	**4.4**	**5**	**4**	**0.032**

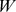
: Body weight (kg), and 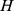: Body height (m),

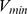
 and 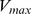: Minimum and maximum gait speed (m/s).

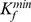
 and 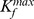: Minimum and maximum quasi-stiffness in flexion stage (Nm/rad).

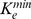
 and 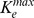: Minimum and maximum quasi-stiffness in extension stage (Nm/rad).

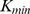
 and 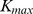: Minimum quasi-stiffness in weight-acceptance phase (Nm/rad).

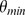
 and 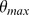 : Minimum and maximum knee excursion in weight-acceptance phase (deg).

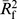
: Average 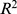 of the linear fit on moment-angle curve in flexion stage.

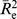
: Average 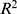 of the linear fit on moment-angle curve in extension stage.

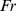
: Froude number.

‡ : Data collected at Human PoWeR Lab, NC State University [24].

† : Data collected at Biomechanics Lab, East Carolina University [29].

First, for each subject, we plotted the knee moment and angle data against each other, (see Fig. 1-bottom for an example gait cycle). The onset of the flexion stage was identified as the point of minimum moment after the heel contacts the ground (point *a*), the end of flexion stage as the point of maximum moment (point *b*), and the end of extension stage as the point of minimum moment before the toe leaves the ground (point *c*). In other words, the flexion stage is composed of the data points between *a* and *b*; and the extension stage between *b* and *c*. Then we applied linear fits between the angle and moment data points in flexion and extension stages (as described in the previous section). The slopes of the fits were correspondingly reported as 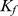 and 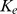, and the average was calculated as 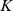. The knee angle at point *a* was subtracted from the angle at point *b* to obtain 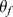; similarly for 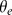 using points *b* and *c*. We averaged 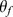 and 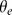 to obtain 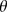.

The inverse dynamics analysis of the previous section proposed three sets of collinear predictors for the models of 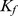, 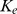, and 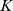. Since, the purpose of this study was to constitute predictive models for 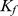, 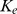, and 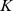 that are composed of these collinear predictors, we cross-validated the models structures. We removed the gait cycles of one subject at a time (stratified cross-validation) from the data pool and conducted Partial Least Square (PLS) analysis to evaluate the predictability of the predictors (i.e. parameters suggested in the previous section). For the sake of completeness, we have reported the optimal number of components that could best describe the response variables (i.e. quasi-stiffnesses) and result in minimal PRESS statistics, in Table 2 [53]-[55]. Next, based on the identified factors of equations (8-a to 8-c), we evaluated these combinations for the 136 gait trials and respectively applied linear regression between them and the values of 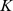, 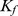, and 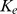. We used least square regression because 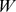 and 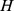 would be known for a specific subject, and 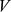 and excursion of the knee are also assumed to be available through measurements taken from corresponding sensors on-board the user or a wearable device. In each case, stepwise, non-significant terms (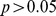) of the regressed polynomial were iteratively removed until we reached *general-form statistical models* that best explain the knee quasi-stiffnesses and that only include the significant parameters.

**Table 2:** General-Form Models to Predict the Quasi-Stiffness of the Knee Joint in Stance for Normal Walking.

Phase	Model	Unit	Error	PLS-CV #Comp.	PLS-CV 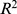	PLS-CV Predicted 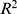	Fit Quality
Flexion	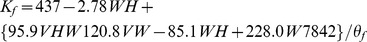	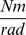	10%	7	88.3%	75.1%	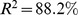 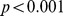
Extension	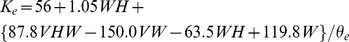	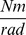	10%	3	83.2%	73.6%	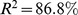 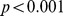
Stance	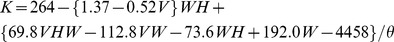	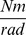	11%	2	75.0%	59.8%	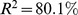 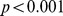

### Stature-Based Models

It is preferred to use the non-dimensional Froude number (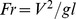, where *l* is the leg length and *g* is the gravitational constant) when working with subjects with different body size [49]. To relate the preferred walking speed to the subject’s stature (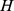 and 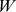), we assume that at the preferred walking speed 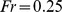 [49]-[52]. We assume an anthropometric relationship of 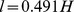 [46]. Thus, the optimal or “preferred” gait speed is approximated as:
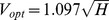
(9)


To exclude the knee excursion from the general-form models, we merely substituted the mean values over the data set (i.e. 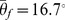, 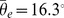, and 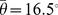) into the general-form models. The reason is twofold: a. the general-form models did not show high dependence on the knee excursion, and b. the knee excursion did not demonstrate high variability around the optimal gait speed of 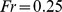 (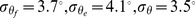). We then applied equation (9) and the average values in the general-form expressions to obtain a series of *stature-based models* intended to predict the quasi-stiffnesses of the knee at the preferred gait speed only as functions of 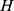 and 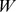.

## Results

The knee demonstrated approximately linear behavior in both flexion and extension stages of stance for nearly all subjects across all gait speeds. Linear fits (similar to that shown in Fig. 1-bottom) demonstrated an average 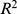 of 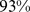 in the flexion stage, and 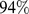 in the extension (Table 1). For each subject, the minimum and maximum values of the knee joint quasi-stiffness (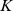) and the knee joint excursion during stance (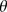) as well as the average values of 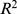 are reported in Table 1. Knee quasi-stiffnesses ranged from a minimum value of 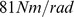 for subject 7 in the extension phase of walking at 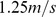 to a maximum value of  for subject 14 in the flexion stage of walking at 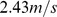 for the gait trials examined here. The average values of 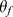, 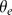, and 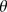 were respectively calculated as 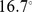, 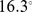, and 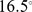.

As Table 2 outlines, the cross-validation analyses suggest 7, 3, and 2 components that can optimally describe 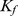, 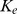, and 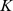 (resulting in minimal PRESS statistics). Table 2 also shows the values of 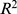 and predicted 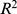 for the PLS analysis. The PLS analyses reconfirms that the predictors that were identified through inverse dynamics analyses can constitute predicting models for 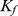, 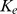, and 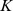. Next, the general-form models were obtained through Least Square Regression as listed in Table 2. We included all the components that the inverse dynamics analysis of Methods Section suggested and removed the components that were not statistically significant. Table 2 lists the general-form models of 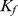, 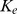, and 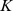.The general-form models are listed in Table 2. Only 1, 5, and 4 data points from 136 trials exhibited outlier behavior in the regression analysis for 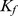, 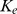, and, 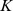, respectively. The values of 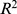 and 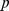 were (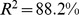, 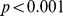) for 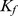, (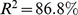, 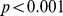) for 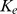, and (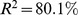, 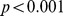) for 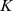, as reported in Table 2. The regression analyses showed 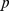-values of 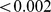 for all of the coefficients of the polynomials, with the exception of 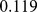 for the intercept of the model polynomial for 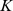 (8-a) and 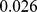 for the coefficient of 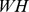 in the model polynomial for 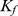 (8-b), implying that the intercept in (8-a) is not significantly greater than zero. The residuals of all three fits were normally distributed and no notable correlation with the order of data collection and magnitude of the quasi-stiffness was observed, except we found slightly greater values for the residuals of the data of subjects 10 to 14 collected at East Carolina University.

**Figure 2:**
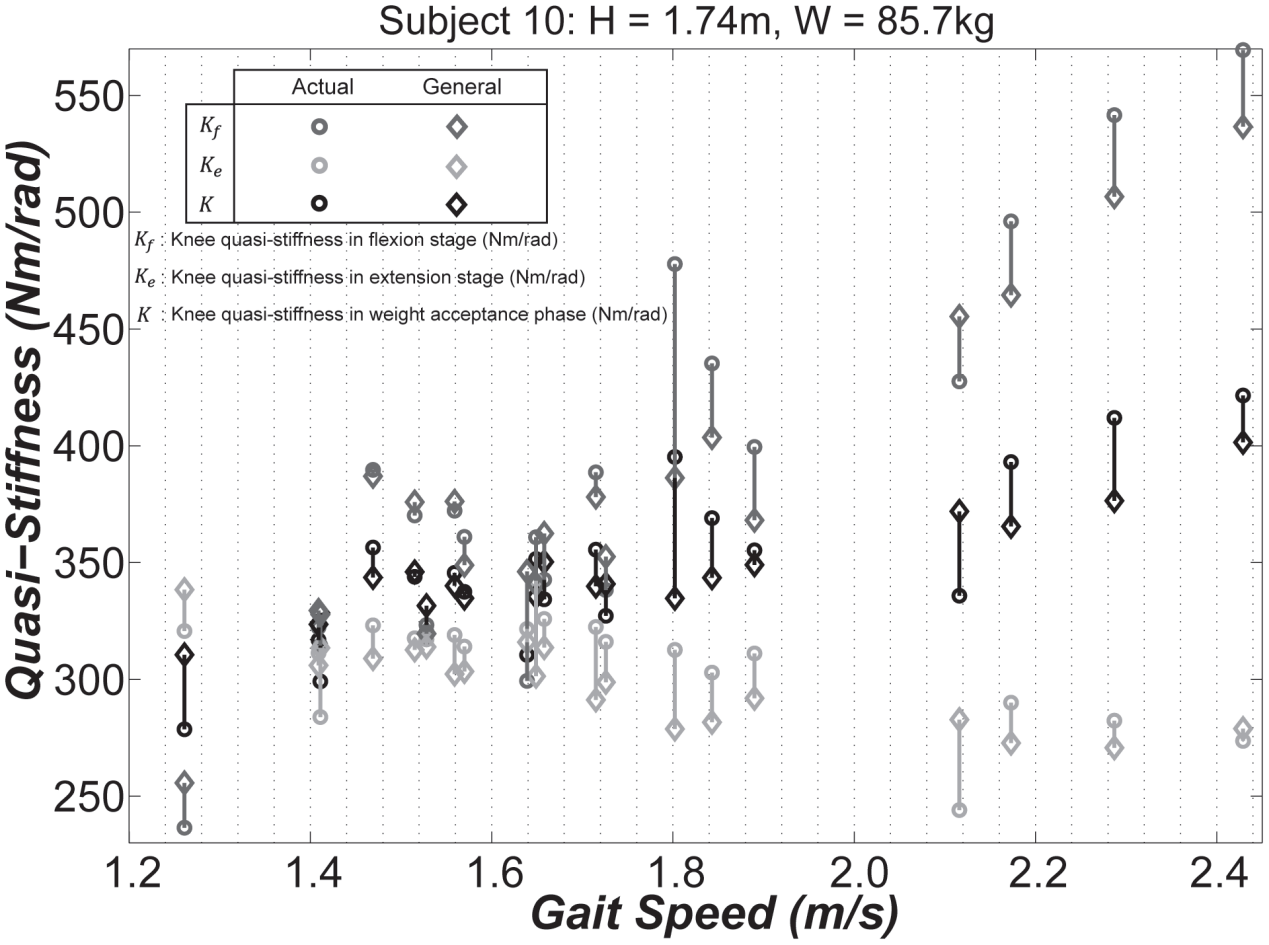
Knee quasi-stiffness for subject 10, as an example, in flexion (dark gray) and extension (light gray) stages, and weight acceptance phase (black) plotted against the gait speed. The circles indicate the experimental values and the diamonds are the predictions of the general-form models (Table 2).

Fig. 2 shows the predictions of general-form models for one of the subjects with 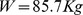 and 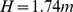 close to the average adults. In this figure, both experimental data, and results of the general-form models are displayed. We observe that 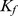 increases as the gait speed increases; whereas, 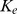 displays a moderate decrease. We also observe that 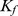 and 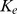 are nearly identical at 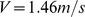, which corresponds to 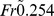. We observed similar phenomenon for all of the subjects. Indeed, 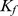 and 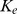 tend to be closest at an average gait speed of 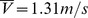 with standard deviation of 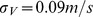 across the subjects, which corresponds to an average Froude number of 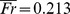 and standard deviation of 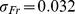. Table 1 lists the values of 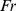 for each subject at which 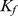 and 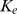 are closest.

**Figure 3:**
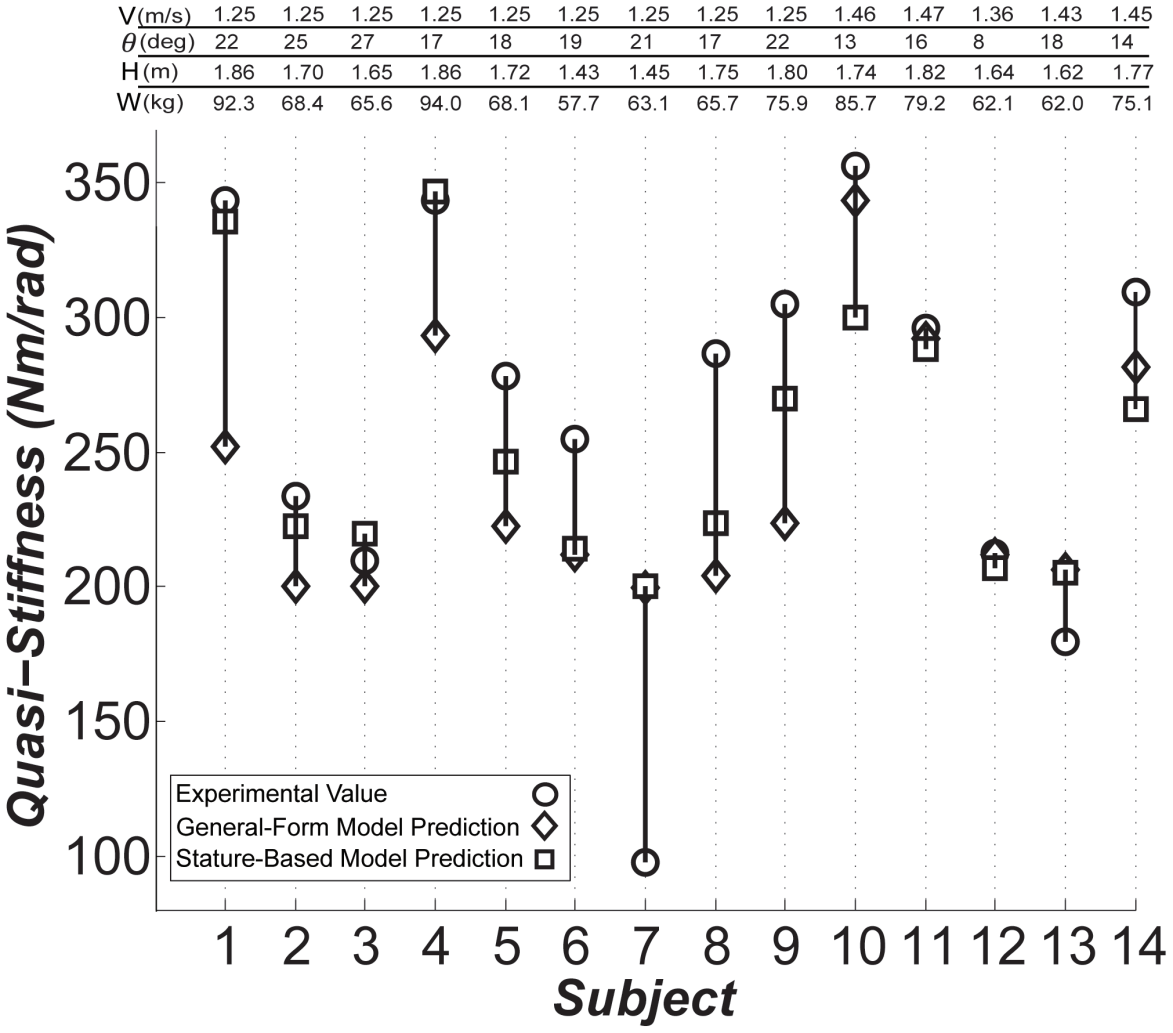
The knee quasi-stiffness in the weight acceptance phase of the gait. The experimental values are shown by circles, and the predictions of the general-form model by diamonds with average error of 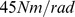 *(14%)*, and the stature-based models by squares with average error of 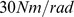 *(9%)* for the optimal gait speed.

The stature-based models are reported in Table 3. Since we do not know the “true” optimal gait speed for each subject, we cannot report 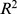 for the models predictions. Instead, we calculated 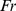 for each gait trial and chose the trial with the speed that is closest to 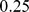 for each subject. These trials are shown in Fig. 3. Subject 7 exhibited outlier behavior of some sort. Our analysis demonstrates that the simplest (stature-based) models predict 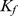 , 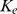 , and 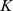 with an average errors of 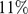, 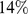, and 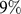 excluding the outlier number 7 (as reported in Table 3), and an average error of 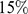, 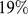, and 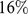 including it.

**Table 3:** Stature-Based Models to Predict the Quasi-Stiffness of the Knee Joint in Stance for Normal Walking at Optimal Gait Speed.

Phase	Model	Unit	Error	Conditions
Flexion	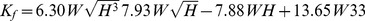	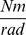	11%	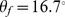 and 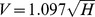
Extension	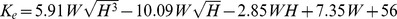	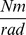	14%	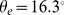 and 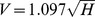
Stance	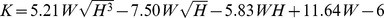	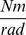	9%	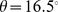 and 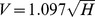

## Discussion

In this paper we have established statistical models that can estimate the quasi-stiffnesses of the knee during stance phase of human walking. To obtain the models, we extracted the generic equation of the knee moment through an inverse dynamics analysis and simplified it for the stance phase. The simplified equation for the stance phase emphasizes that the quasi-stiffness of the knee is correlated with linear combinations of both gait and body parameters (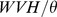, 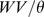, 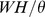, 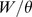, 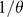, 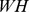, and 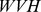) in its most general form. Using a data set spanning a relatively wide range of speeds and body sizes, we constituted expressions that statistically best describe the quasi-stiffness of the knee in the flexion and extension stages, and the entire weight acceptance phase of stance. In addition, we developed more simplified (and perhaps practical) models that are independent of knee excursion and gait speed.

We found high values of 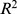 for linear curve fits to the moment-angle relationship at the knee in both the flexion and extension stages (as shown in Table 1) that are in good agreement with previous results [30], [34]. We observed that the knee exhibits identical quasi-stiffness in the flexion and extension stages (*spring-type behavior*) at the non-dimensional gait speed of 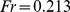. At other gait speeds, the knee still exhibits linear behavior (more compliant at slow speeds than at fast speeds) in both flexion and extension stages but with different equilibrium angles, which implies non-zero mechanical energy expenditure (the trend of mechanical work change vs. gait speed is shown elsewhere [34]). This finding is in accordance with the results of other researchers who showed that the rate of energy recovery is highest when the subject is walking with the preferred gait speed [49], [56].

From a design point of view, our results suggest that a device (including orthoses, exoskeletons, prostheses, and biped robots) can approximate the behavior of the human knee by utilizing a spring with stiffness equal to the quasi-stiffness of the knee at the preferred gait speed. For other gait speeds, the stiffness of the device might ideally be tuned based on the equations presented in Table 2. For this purpose, the device would in a real-time mode measure the gait speed (e.g. using a GPS), knee excursion (e.g. using a goniometer), and weight. However, since realization of a variable stiffness mechanism is difficult to achieve, the net quasi-stiffness of the weight acceptance phase (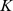) might be a viable alternative for the spring constant of the envisioned device. As such, the knee might be approximately modeled by a single torsional spring with stiffness equal to the mean of the stiffness of the flexion and extension stages at the preferred gait speed of 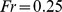. This is a reasonable choice for two reasons: 1) humans prefer to walk with a speed that is dictated by their body size [*49*]–[*52*], and 2) 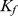 and 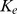 tend to be identical at the preferred gait speed and deviate at lower and higher speeds. This reemphasizes the results of our previous work [34] where we showed that the stiffness, angle of engagement, and amount of rotation of the device joint should be deliberately chosen based on the gait parameters.

Recently, researchers in the field of prosthetics have moved toward quasi-passive systems and implemented impedance control methods in their designs [7], [43], [57], [58]. In most design application, the kinetic and kinematic data for the target users are not available. However, sizing orthoses and prostheses requires *a priori* knowledge of the knee quasi-stiffness variability for the users. To size the stiffness of the prosthetic and orthotic devices, the designers utilize the average quasi-stiffness extracted from the kinetic and kinematic data of sample healthy subjects [7], [9], [17], [43], [59]. The stiffness that designers use range from ∼50 *Nm/rad* to ∼430 *Nm/rad*, depending on the sample population that the designers have chosen and the tuning process [7], [9], [43]. The sample population is usually composed of individuals with weight, height, and preferred gait speed that are not necessarily representatives of the target user.

To examine the differences between a model that is based on the average data and the models developed here, we found the average values of 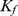, 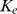, and 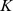 for the gait data utilized in our study and examined the error between the average quasi-stiffnesses and the true subject-specific quasi-stiffnesses. Table 4 compares the average error associated with the general-form models, stature-based models, and a model that merely uses the average values of 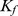, 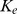, and 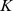 (as reported in Table 1). The results show much larger errors when the average values are utilized than with our models. Therefore, we hypothesize that selection of the device stiffness based on the models presented here would result in a more natural and user/gait-adaptable performance for the knee orthoses and prostheses. All together, the models developed in this study may help researchers and clinicians tune the stiffness of knee orthoses and prostheses according to the body size and gait speed of the user, and do so without requiring to perform additional subject-specific gait analyses.

**Table 4:** Average Error Values for Different Models.

Parameter	General-Form	Stature-Based	Average Values
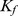	10%	11%	32%
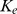	10%	14%	27%
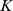	11%	9%	24%

Applications of the models presented in this study are not restricted to the field of medical orthoses and prostheses. These models could also be used for the design of knee exoskeletons that are meant to augment the performance of a healthy knee. Researchers have proposed a range of sophistication in the design of exoskeletons from quasi-passive to fully active systems [3], [4], [6]. Our findings suggest that passive components (i.e. springs) could be further exploited in the design of these devices; provided that the passive components are properly tuned for the gait and user. In fact, the design models of Table 3 suggest that the stiffness of an assistive device should ideally be adapted based on the weight and height of the subject.

Our study had a few limitations worth noting. First, we only addressed the behavior of the knee during stance phase of normal walking on level ground. Our approach could be extended to other joints of the lower-limb, other gait regimes (e.g. running) and also account for variable terrain or carried loads. For example, the quasi-stiffness of the ankle significantly increases as the ground slope changes [35]; similarly, we anticipated that the quasi-stiffness of the knee might also be tuned on uneven ground.

Another limitation was that in order to establish the current models, we used 136 gait trials for 14 adult subjects. Therefore, our analyses could be generalized only to the range of age, height, weight, and gait speed that the subjects represent and as much as the statistical significance supports. Similar statistical analyses could be carried out on other groups of subjects such as children or older adults and other locomotion regimes such as running to establish similar models. We employed several simplification and estimation steps to identify the important predictors that only hold when the subject walks on the sagittal plane with no pathologies in the gait. A more sophisticated model could take the eliminated terms and confined parameters into account. For example, researchers have shown significant dependence of the ankle quasi-stiffness on the gender and age [32]; similar phenomena might be expected for the knee.

Taken together, we have established a family of models with different levels of sophistication that predict the quasi-stiffnesses of the knee in stance. From an applied standpoint, our models could be used in gait analysis, modeling, and simulations, and also in the fields of orthotics, prosthetics, and bipedal robots.

## Supporting Information

Figure S1
**A schematic model of the support shank and foot for a subject walking on the sagittal plane.** The figure depicts the proximal force and moments of the shank and foot segments, and the center of masses (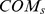 and 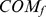). The ground reaction force and moment are also shown at the center of pressure (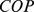).(TIF)

Table S1 **Description of mathematical expressions.**
(DOCX)

Appendix S1 **Inverse dynamics analysis.**
(DOCX)
