# Supplementary figures and images for: Estimation of Quasi-Stiffness of the Human Knee in the Stance Phase of Walking
Source: PLoS One. 2013 Mar 22;8(3):e59993. doi: 10.1371/journal.pone.0059993 (PMC3606171; doi:10.1371/journal.pone.0059993)

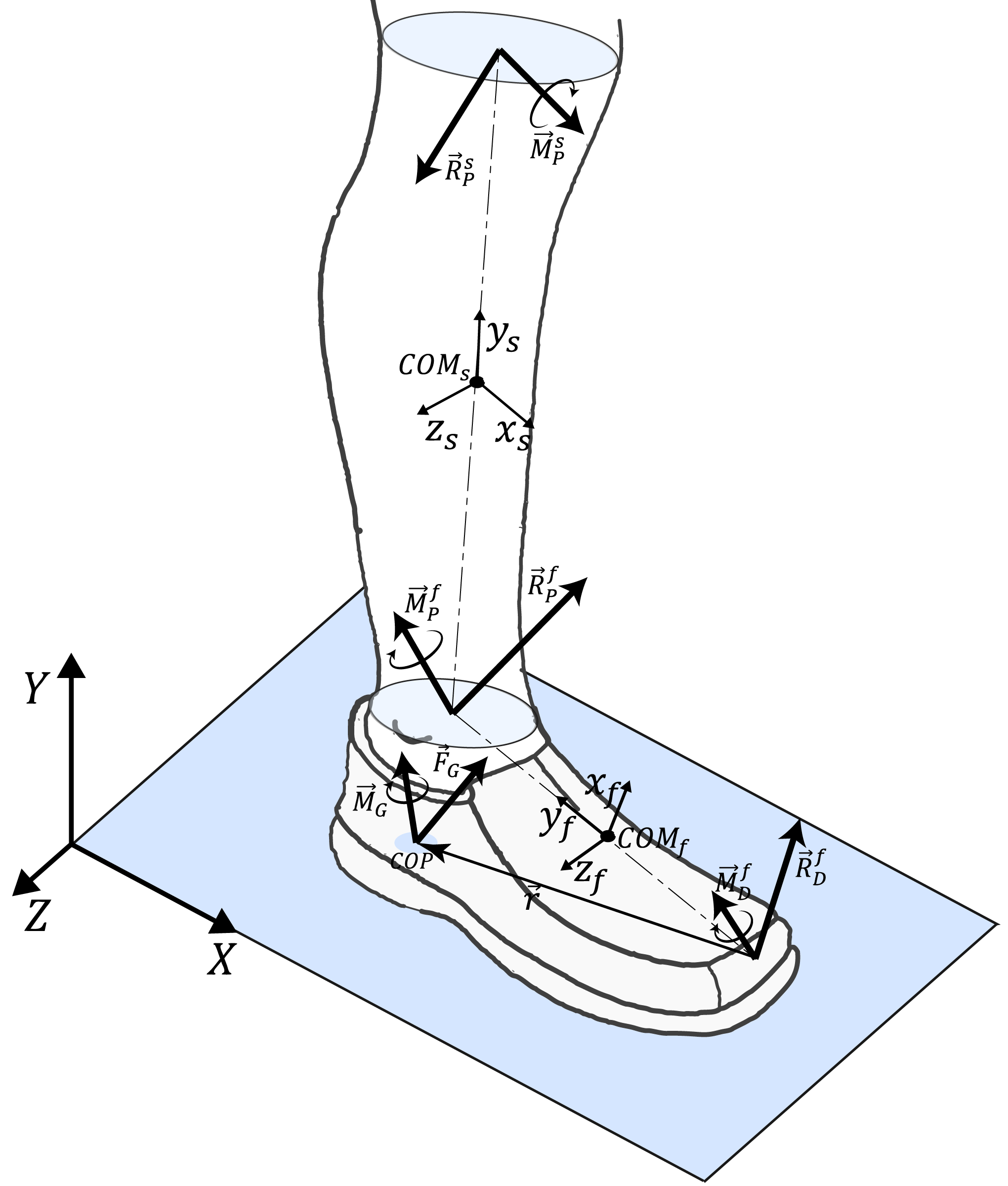

Supplement: Figure S1 — A schematic model of the support shank and foot for a subject walking on the sagittal plane. The figure depicts the proximal force and moments of the shank and foot segments, and the center of masses ( and ). The ground reaction force and moment are also shown at the center of pressure (). (TIF) [file pone.0059993.s001.tif]
