# Supplementary material for: Estimation of Quasi-Stiffness of the Human Knee in the Stance Phase of Walking
Source: PLoS One. 2013 Mar 22;8(3):e59993. doi: 10.1371/journal.pone.0059993 (PMC3606171; doi:10.1371/journal.pone.0059993)
Supplement: Table S1 — Description of mathematical expressions. (DOCX) [file pone.0059993.s002.docx]

**TABLE S1.** Description of the Mathematical Expressions

| **Parameter** | **Description** | **Parameter** | **Description** |
| --- | --- | --- | --- |
| $\boldsymbol{K}_{\boldsymbol{f}}$ | Knee quasi-stiffness in flexion stage of stance | ${\vec{\boldsymbol{U}}}_{\boldsymbol{f}}$ | Angular momentum of foot |
| $\boldsymbol{K}_{\boldsymbol{e}}$ | Knee quasi-stiffness in extension stage of stance | ${\vec{\boldsymbol{U}}}_{\boldsymbol{s}}$ | Angular momentum of shank |
| $\boldsymbol{K}$ | Knee quasi-stiffness in weight acceptance phase | $\left[ \boldsymbol{I}_{\boldsymbol{f}} \right]$ | Matrix of moment of inertia of foot |
| $\boldsymbol{\theta}_{\boldsymbol{f}}$ | Knee excursion in flexion stage | $\left[ \boldsymbol{I}_{\boldsymbol{s}} \right]$ | Matrix of moment of inertia of shank |
| $\boldsymbol{\theta}_{\boldsymbol{e}}$ | Knee excursion in extension stage | ${\vec{\boldsymbol{M}}}_{\boldsymbol{P}}^{\boldsymbol{s}}$ | Shank proximal moment in global coordinate system |
| $\boldsymbol{\theta}$ | Knee excursion in weight acceptance phase | ${\vec{\boldsymbol{M}}}_{\boldsymbol{D}}^{\boldsymbol{s}}$ | Shank distal moment in global coordinate system |
| $\boldsymbol{W}$ | Body weight | ${\vec{\boldsymbol{M}}}_{\boldsymbol{P}}^{\boldsymbol{f}}$ | Foot proximal moment in global coordinate system |
| $\boldsymbol{V}$ | Gait speed | ${\vec{\boldsymbol{M}}}_{\boldsymbol{D}}^{\boldsymbol{f}}$ | Foot distal moment in global coordinate system |
| $\boldsymbol{H}$ | Body height | ${\vec{\boldsymbol{M}}}_{\boldsymbol{p}}^{\boldsymbol{s}}$ | Shank proximal moment in shank anatomical coordinate system |
| ${\vec{\boldsymbol{M}}}_{\boldsymbol{G}}$ | Ground reaction moment | ${\vec{\boldsymbol{M}}}_{\boldsymbol{d}}^{\boldsymbol{s}}$ | Shank distal moment in shank anatomical coordinate system |
| ${\vec{\boldsymbol{F}}}_{\boldsymbol{G}}$ | Ground reaction force | ${\vec{\boldsymbol{M}}}_{\boldsymbol{p}}^{\boldsymbol{f}}$ | Foot proximal moment in shank anatomical coordinate system |
| $\vec{\boldsymbol{r}}$ | Vector from toe to center of pressure | ${\vec{\boldsymbol{M}}}_{\boldsymbol{d}}^{\boldsymbol{f}}$ | Foot distal moment in shank anatomical coordinate system |
| $\boldsymbol{L}_{\boldsymbol{f}}$ | Foot length | ${\vec{\boldsymbol{R}}}_{\boldsymbol{P}}^{\boldsymbol{s}}$ | Shank proximal force in global coordinate system |
| $\boldsymbol{L}_{\boldsymbol{s}}$ | Shank length | ${\vec{\boldsymbol{R}}}_{\boldsymbol{D}}^{\boldsymbol{s}}$ | Shank distal force in global coordinate system |
| ${\bar{\boldsymbol{e}}}_{\boldsymbol{Y}}^{\boldsymbol{f}}$ | Unit vector along foot segment | ${\vec{\boldsymbol{R}}}_{\boldsymbol{P}}^{\boldsymbol{f}}$ | Foot proximal force in global coordinate system |
| ${\bar{\boldsymbol{e}}}_{\boldsymbol{Y}}^{\boldsymbol{s}}$ | Unit vector along shank segment | ${\vec{\boldsymbol{R}}}_{\boldsymbol{D}}^{\boldsymbol{f}}$ | Foot distal force in global coordinate system |
| $\boldsymbol{m}_{\boldsymbol{f}}$ | Foot mass | ${\vec{\boldsymbol{R}}}_{\boldsymbol{p}}^{\boldsymbol{s}}$ | Shank proximal force in shank anatomical coordinate system |
| $\boldsymbol{m}_{\boldsymbol{s}}$ | Shank mass | ${\vec{\boldsymbol{R}}}_{\boldsymbol{d}}^{\boldsymbol{s}}$ | Shank distal force in shank anatomical coordinate system |
| ${\vec{\boldsymbol{a}}}_{\boldsymbol{f}}$ | Foot acceleration | ${\vec{\boldsymbol{R}}}_{\boldsymbol{p}}^{\boldsymbol{f}}$ | Shank proximal force in shank anatomical coordinate system |
| ${\vec{\boldsymbol{a}}}_{\boldsymbol{s}}$ | Shank acceleration | ${\vec{\boldsymbol{R}}}_{\boldsymbol{d}}^{\boldsymbol{f}}$ | Shank distal force in shank anatomical coordinate system |
| $\boldsymbol{g}$ | Magnitude of acceleration due to gravity | $\boldsymbol{X-Y-Z}$ | Global coordinate system |
| $\boldsymbol{L}_{\boldsymbol{p}}^{\boldsymbol{f}}$ | Distance between center of mass of foot to ankle | $\boldsymbol{x}_{\boldsymbol{s}}\boldsymbol{-}\boldsymbol{y}_{\boldsymbol{s}}\boldsymbol{-}\boldsymbol{z}_{\boldsymbol{s}}$ | Anatomical coordinate system of shank |
| $\boldsymbol{L}_{\boldsymbol{p}}^{\boldsymbol{s}}$ | Distance between center of mass of shank to knee | $\boldsymbol{x}_{\boldsymbol{f}}\boldsymbol{-}\boldsymbol{y}_{\boldsymbol{f}}\boldsymbol{-}\boldsymbol{z}_{\boldsymbol{f}}$ | Anatomical coordinate system of foot |
| $\left[ \boldsymbol{AG} \right]_{\boldsymbol{s}}$ | Transformation matrix from anatomical system of shank to global system | $\boldsymbol{M}_{\boldsymbol{K}}^{\boldsymbol{Z}}$ | Knee moment on the sagittal plane |
| $\left[ \boldsymbol{GA} \right]_{\boldsymbol{s}}$ | Transformation matrix from global system to anatomical system of shank | ${\vec{\boldsymbol{d}}}_{\boldsymbol{f}}$ | Vector connecting center of mass of foot to toe |
| $\left[ \boldsymbol{AG} \right]_{\boldsymbol{f}}$ | Transformation matrix from anatomical system of foot to global system | ${\vec{\boldsymbol{p}}}_{\boldsymbol{f}}$ | Vector connecting center of mass of foot to ankle |
| $\left[ \boldsymbol{GA} \right]_{\boldsymbol{f}}$ | Transformation matrix from global system to anatomical system of foot | ${\vec{\boldsymbol{d}}}_{\boldsymbol{s}}$ | Vector connecting center of mass of shank to ankle |
| ${\vec{\boldsymbol{\omega}}}_{\boldsymbol{s}}$ | Angular velocity of shank | ${\vec{\boldsymbol{p}}}_{\boldsymbol{s}}$ | Vector connecting center of mass of shank to knee |
| ${\vec{\dot{\boldsymbol{\omega}}}}_{\boldsymbol{s}}$ | Angular acceleration of shank | ${\vec{\boldsymbol{F}}}_{\boldsymbol{s}}$ | Any force applied on shank |
| ${\vec{\boldsymbol{\omega}}}_{\boldsymbol{f}}$ | Angular velocity of foot | ${\vec{\boldsymbol{F}}}_{\boldsymbol{f}}$ | Any force applied on foot |
| ${\vec{\dot{\boldsymbol{\omega}}}}_{\boldsymbol{f}}$ | Angular acceleration of foot | ${\vec{\boldsymbol{M}}}_{\boldsymbol{s}}$ | Any moment applied on shank |
| $\boldsymbol{COM}_{\boldsymbol{s}}$ | Center of mass of shank | ${\vec{\boldsymbol{M}}}_{\boldsymbol{f}}$ | Any moment applied on foot |
| $\boldsymbol{COM}_{\boldsymbol{f}}$ | Center of mass of foot | $\boldsymbol{Fr}$ | Froude number for walking |
| ${\bar{\boldsymbol{e}}}_{\boldsymbol{Y}}$ | Unit vector vertical to the ground and along $Y$ |  | |
