# Supplementary material for: Estimation of Quasi-Stiffness of the Human Knee in the Stance Phase of Walking
Source: PLoS One. 2013 Mar 22;8(3):e59993. doi: 10.1371/journal.pone.0059993 (PMC3606171; doi:10.1371/journal.pone.0059993)
Supplement: Appendix S1 — Inverse dynamics analysis. (DOCX) [file pone.0059993.s003.docx]

# **APPENDIX: INVERSE DYNAMICS ANALYSIS**

In this section, we derive the equations of the reaction forces and moments for the ankle and knee joints. We are mainly interested in the expression of the knee moment. Winter presents a detailed explanation of the inverse dynamics analysis [46] . Our analyses develop the Newtonian equations of motion of foot with respect to (w.r.t.) the global coordinate system as shown by $X-Y-Z$ in Fig. S1. Next, we derive an expression for the moment of the ankle joint w.r.t. the anatomical coordinate frame of the foot shown by $x_{f}-y_{f}-z_{f}$ in Fig. S1. The anatomical coordinate frame of the foot is established by placing $y_{f}$ on the axis connecting the toe to the ankle center, $z_{f}$ along with $Z$ and $x_{f}$ along with the cross-product of $y_{f}$ and $z_{f}$. The Newtonian equations of motion of the shank give us the reaction force of the knee in the global axes, using the reaction force of the ankle. Then, we apply the reaction forces of the ankle and knee in the Euler equations of motion and derive an expression for the moment of the knee w.r.t. the anatomical coordinate frame of the shank shown by $x_{s}-y_{s}-z_{s}$. The anatomical coordinate frame of the shank is established by placing $y_{s}$ on the axis connecting the ankle center to the knee center, $z_{s}$ along $Z$ and $x_{s}$ along the cross-product of $y_{s}$ and $z_{s}$. Table S1 lists the parameters that are used in this text and brief description of each parameter.

The pair of ground reaction force ($\vec{F}_{G}$) and ground reaction moment ($\vec{M}_{G}$) is transferred to the distal joint (i.e. toe) from the center of pressure (COP) to obtain the distal force ($\vec{R}_{D}^{f}$) and the distal moment ($\vec{M}_{D}^{f}$) of the foot w.r.t. $X-Y-Z$. Hence w.r.t. $X-Y-Z$ we get:

$\vec{R}_{D}^{f}=\vec{F}_{G}$ (A-1-a)

$\vec{M}_{D}^{f}=\vec{M}_{G}-\vec{F}_{G}\times\vec{r}$ (A-1-b)

and w.r.t. $x_{f}-y_{f}-z_{f}$ we get:

$\vec{R}_{d}^{f}=\left[GA\right]_{f}\vec{F}_{G}$ (A-2-a)

$\vec{M}_{d}^{f}=\left[GA\right]_{f}\left(\vec{M}_{G}-\vec{F}_{G}\times\vec{r}\right)$ (A-2-b)

$\vec{r}$ is the vector connecting the distal joint to COP and $\left[GA\right]_{f}$ is a proper (i.e. reserves inner product and has a determinant of 1) rotation from $X-Y-Z$ to $x_{f}-y_{f}-z_{f}$. The reaction force at the ankle ($\vec{R}_{P}^{f}$) is derived using the Newtonian equation of motion for the foot:

$\sum^{ } \vec{F}_{f}=m_{f}\vec{a}_{f} \therefore\vec{R}_{P}^{f}=\vec{R}_{D}^{f}+m_{f}\vec{a}_{f}+m_{f}g\bar{e}_{Y}$ (A-3)

where, $\vec{F}_{f}$ denotes any force that is applied on the foot segment and $\vec{a}_{f}$ is the acceleration of the center of mass of the foot ($COM_{f}$). $m_{f}$ is the mass of the foot segment, $\bar{e}_{Y}$ is a unit vector along the *Y*-axis, and *g* is the acceleration due to gravity. Equation (A-1-a) gives us the proximal force of the foot w.r.t. $X-Y-Z$ as:

$\vec{R}_{P}^{f}=\vec{F}_{G}+m_{f}\vec{a}_{f}+m_{f}g\bar{e}_{Y}$ (A-4)

which could be transformed to $x_{f}-y_{f}-z_{f}$ through a rotation, as:

$\vec{R}_{p}^{f}=\left[GA\right]_{f}\vec{R}_{P}^{f}$ (A-5)

Now, we develop the Euler equation of motion for the foot segment w.r.t. $x_{f}-y_{f}-z_{f}$ to derive the expression of the moment at its proximal joint (i.e. ankle):

(A-6)

wherein, $\vec{M}_{f}$ denotes any moment that is applied on the foot segment and $\left[I_{f}\right]$ is the matrix of moment of inertia. $\vec{\omega}_{f}$ the angular velocity, the angular acceleration, and $\vec{U}_{f}$ is the angular momentum of the foot segment. Expanding the left hand side of the above equation gives us:

(A-7)

where,$\vec{M}_{p}^{f}$is the moment at the proximal joint of the foot segment expressed w.r.t. $x_{f}-y_{f}-z_{f}$. $\vec{d}_{f}$ is the vector that connects the center of mass of the foot ($COM_{f}$) to the toe and $\vec{p}_{f}$ is the vector that connects $COM_{f}$ to the ankle joint both expressed w.r.t. $x_{f}-y_{f}-z_{f}$. Here, the tip of the toe is chosen such that $COM_{f}$ relies on the origin of $x_{f}-y_{f}-z_{f}$ Now, we insert the corresponding terms in equation (A-7):

(A-8)

Since $\left[GA\right]_{f}$ is a proper rotation, $\vec{M}_{p}^{f}$ could be transformed into $X-Y-Z$ as:

(A-9)

where, $\left[AG\right]_{f}=\left[GA\right]_{f}^{-1}$. One should notice that:

$\vec{F}_{G}\times L_{f}\bar{e}_{Y}^{f}= -\vec{F}_{G}\times\left[AG\right]_{f}\vec{d}_{f}+\vec{F}_{G}\times\left[AG\right]_{f}\vec{p}_{f}$ (A-10)

where, $L_{f}$ is the length of the foot segment and $\bar{e}_{Y}^{f}$ is the unit vector along the $y_{f}$-axis of the foot segment expressed w.r.t. Y-axis. Thus, equation (A-9) can be written in the following form:

(A-11)

where, $L_{p}^{f}$ is the distance between $COM_{f}$ and the ankle.

Next, we derive the proximal moment of the shank segment (i.e. knee moment). We exploit the expressions of the proximal force and moment for the foot segment and use them as the distal force ($\vec{R}_{D}^{s}$) and moment ($\vec{M}_{D}^{s}$) of the shank. In other words w.r.t. $X-Y-Z$ we have:

$\vec{R}_{D}^{s}=\vec{R}_{P}^{f}$ (A-12-a)

$\vec{M}_{D}^{s}=\vec{M}_{P}^{f}$ (A-12-b)

which can be transformed to $x_{s}-y_{s}-z_{s}$ using the *proper rotation*-matrix of $\left[GA\right]_{s}$:

$\vec{R}_{d}^{s}=\left[GA\right]_{s}\vec{R}_{P}^{f}$ (A-13-a)

$\vec{M}_{d}^{s}=\left[GA\right]_{s}\vec{M}_{P}^{f}$ (A-13-b)

The proximal reaction force of the shank ($\vec{R}_{P}^{s}$) is obtained from the Newtonian equation of motion w.r.t. $X-Y-Z$, as follows:

$\sum^{ } \vec{F}_{s}=m_{s}\vec{a}_{s} \therefore\vec{R}_{P}^{s}=\vec{R}_{D}^{s}+m_{s}\vec{a}_{s}+m_{s}g\bar{e}_{Y}$ (A-14)

where,$\vec{F}_{s}$ denotes any force that is applied on the shank segment, $m_{s}$ is the mass and $\vec{a}_{s}$ is the acceleration of the center of mass ($COM_{s}$) for the shank segment. Applying equations (A-4-a) and (A-12-a) in (A-14), we get:

$\vec{R}_{P}^{s}=\vec{F}_{G}+\left(m_{f}\vec{a}_{f}+m_{s}\vec{a}_{s}\right)+\left(m_{f}+m_{s}\right)g\bar{e}_{Y}$ (A-15)

which could be transformed to $x_{s}-y_{s}-z_{s}$ as:

$\vec{R}_{p}^{s}=\left[GA\right]_{s}\vec{R}_{P}^{s}$ (A-16)

Now, we employ the Euler equation of motion for the shank w.r.t. $x_{s}-y_{s}-z_{s}$ to derive the moment at the proximal joint (i.e. knee). As such:

(A-17)

where, $\vec{M}_{s}$ denotes any moment that is applied on the shank. $\left[I_{s}\right]$ is the matrix of moment of inertia, $\vec{\omega}_{s}$ the angular velocity, is the angular acceleration, and $\vec{U}_{s}$ is the angular momentum for the shank segment. Expanding the left hand side of the above equation results in:

(A-18)

where, $\vec{M}_{p}^{s}$ is the moment at the proximal joint of the shank (i.e. knee moment). $\vec{d}_{s}$ is the vector connecting the center of mass of the shank ($COM_{s}$) to the ankle and $\vec{p}_{s}$ the vector connecting $COM_{s}$ to the knee both expressed in $x_{s}-y_{s}-z_{s}$ . Inserting the corresponding expression of each term in (A-18) concludes:

$$-\left[GA\right]_{s}\left(\vec{F}_{G}+m_{f}\vec{a}_{f}+m_{f}g\bar{e}_{Y}\right)\times\vec{d}_{s}+\left[GA\right]_{s}\left(\vec{F}_{G}+\left(m_{f}\vec{a}_{f}+m_{s}\vec{a}_{s}\right)+\left(m_{f}+m_{s}\right)g\bar{e}_{Y}\right)\times\vec{p}_{s}$$

(A-19)

The proximal moment of the shank ($\vec{M}_{p}^{s}$) is transformed to $X-Y-Z$ to reach the following expression for the knee moment ($\vec{M}_{P}^{s}$):

$$-\left(\vec{F}_{G}+m_{f}\vec{a}_{f}+m_{f}g\bar{e}_{Y}\right)\times\left[AG\right]_{s}\vec{d}_{s}+\left(\vec{F}_{G}+\left(m_{f}\vec{a}_{f}+m_{s}\vec{a}_{s}\right)+\left(m_{f}+m_{s}\right)g\bar{e}_{Y}\right)\times\left[AG\right]_{s}\vec{p}_{s}$$

(A-20)

where, $\left[AG\right]_{s}=\left[GA\right]_{s}^{-1}$. One should notice that:

$\vec{F}_{G}\times L_{s}\bar{e}_{Y}^{s}= -\vec{F}_{G}\times\left[AG\right]_{s}\vec{d}_{s}+\vec{F}_{G}\times\left[AG\right]_{s}\vec{p}_{s}$ (A-21)

where, $L_{s}$ is the length of the shank segment and $\bar{e}_{Y}^{s}$ is a unit vector along $y_{s}$-axis of the shank segment expressed w.r.t. $X-Y-Z$. Hence, we obtain the following expression for the moment of the knee joint:

$$\vec{M}_{P}^{s}=\left\{\vec{M}_{G}-\vec{F}_{G}\times\left(\vec{r}+L_{f}\bar{e}_{Y}^{f}+L_{s}\bar{e}_{Y}^{s}\right)\right\}+$$

$$\left\{\left(m_{f}\vec{a}_{f}+m_{f}g\bar{e}_{Y}\right)\times\left(L_{p}^{f}\bar{e}_{Y}^{f}+L_{s}\bar{e}_{Y}^{s}\right)+\left(m_{s}\vec{a}_{s}+m_{s}g\bar{e}_{Y}\right)\times L_{p}^{s}\bar{e}_{Y}^{s} \right\}+$$

(A-22)

where, $L_{p}^{s}$ is the distance between $COM_{s}$ and the knee.
